# Effect of Surgeon Volume on Mechanical Complications after Resection Arthroplasty with Articulating Spacer

**DOI:** 10.3390/jpm14050490

**Published:** 2024-05-03

**Authors:** Chih-Yuan Ko, Chun-Hao Tsai, Yi-Chin Fong, Hui-Yi Chen, Hsien-Te Chen, Tsung-Li Lin

**Affiliations:** 1 Department of Orthopedics, China Medical University Hospital, Taichung 40447, Taiwan; 014333@tool.caaumed.org.tw (C.-Y.K.); 007940@tool.caaumed.org.tw (C.-H.T.); 001762@tool.caaumed.org.tw (Y.-C.F.); 002326@tool.caaumed.org.tw (H.-T.C.); 2 Graduate Institute of Biomedical Sciences, China Medical University, Taichung 40402, Taiwan; 3 Department of Sports Medicine, College of Health Care, China Medical University, Taichung 40604, Taiwan; 4 Department of Radiology, China Medical University Hospital, Taichung 40447, Taiwan; 007396@tool.caaumed.org.tw

**Keywords:** prosthetic knee infection, high volume, low volume, resection arthroplasty, articulating spacer, mechanical complications

## Abstract

Two-stage revision with an antibiotic-loaded cement articulating spacer is a standard treatment for chronic prosthetic knee infection (PKI); however, mechanical complications can occur during the spacer period. There is limited evidence on the association between surgeon volume and mechanical complications after resection arthroplasty (RA) using an articulating spacer. This study aimed to compare the rates of mechanical complications and reoperation after RA with articulating spacers by surgeons with high volumes (HV) and low volumes (LV) of RA performed and analyzed the risk factors for mechanical failure. The retrospective study investigated 203 patients treated with PKIs who underwent RA with articulating spacers and were divided according to the number of RAs performed by the surgeons: HV (≥14 RAs/year) or LV (<14 RAs/year). Rates of mechanical complications and reoperations were compared. Risk factors for mechanical complications were analyzed. Of the 203 patients, 105 and 98 were treated by two HV and six LV surgeons, respectively. The mechanical complication rate was lower in HV surgeons (3.8%) than in LV surgeons (36.7%) (*p* < 0.001). The reoperation rate for mechanical complications was lower in HV surgeons (0.9%) than in LV surgeons (24.5%) (*p* < 0.001). Additionally, 47.2% of patients required hinge knees after mechanical spacer failure. Medial proximal tibial angle < 87°, recurvatum angle > 5°, and the use of a tibial spacer without a cement stem extension were risk factors for mechanical complications. Based on these findings, we made the following three conclusions: (1) HV surgeons had a lower rate of mechanical complications and reoperation than LV surgeons; (2) mechanical complications increased the level of constraint in final revision knee arthroplasty; and (3) all surgeons should avoid tibial spacer varus malalignment and recurvatum deformity and always use a cement stem extension with a tibial spacer.

## 1. Introduction

Prosthetic knee infections (PKIs) occur in 1–2% of primary total knee arthroplasties (TKA) [1]. In addition, approximately 25% of revision TKAs are performed for PKIs [2]. Furthermore, PKIs increasingly concern arthroplasty surgeons due to their high financial burden on the healthcare system, large negative effect on patient outcomes, and high mortality rates [3].

Acute PKIs may be treated effectively with debridement, antibiotics, and implant retention procedure; however, chronic infections often require a two-stage revision [4]. Two-stage revision with antibiotic-loaded cement articulating spacers is a standard treatment for chronic PKIs, as it provides a greater range of motion and has better functional scores, high infection eradication, and easier revision approaches [5]. Despite their advantages, less than half of articulating spacers are considered optimally sized and positioned, and the rates of various mechanical complications, such as spacer fracture or migration, periprosthetic fracture, joint subluxation or dislocation, or extensor mechanism disruption, as high as 57%, have been reported [6]. These mechanical complications may lead to additional spacer exchange, compromised functional outcomes, prolonged treatment course, the need for more constrained prostheses, and decreased survivorship after reimplantation [7,8].

High-volume (HV) surgeons achieve better results and lower complication rates than low-volume (LV) surgeons in primary TKAs [9]. Similarly, HV surgeons have better outcomes and lower re-revision rates following aseptic revision TKA than LV surgeons, and they support the development of revision teams within arthroplasty centers to offer patients the best outcomes [10]. Furthermore, establishing a specialized PKI center with experienced surgeons performing HV infection procedures is recommended to improve the rate of reimplantation [11]. However, the effects of surgeon volume on mechanical complications following resection arthroplasty (RA) using articulating spacers remain unclear. To the best of our knowledge, only one study found that being an LV surgeon is a risk factor associated with dislocation and malalignment/malpositioning of spacers [8]. Understanding the outcomes of HV and LV surgeons is helpful in counseling patients and taking measures to improve the success of two-stage revisions.

Therefore, this retrospective study had the dual aims of comparing the rate of mechanical complications and reoperation after RA with articulating spacers by HV or LV surgeons and analyzing the risk factors for mechanical failure. We hypothesized that HV surgeons would have fewer mechanical complications and reoperations.

## 2. Material and Methods

### 2.1. Articulating Spacers

Posterior-stabilized (PS) cement articulating spacers were used (CADAS; EverYoung BioDimensions, Taichung, Taiwan) [12]. A PS spacer with a post-cam construct was fabricated intraoperatively using a silicone mold. There are six sizes of femoral and tibial trials and silicone molds available, with sizes increasing from 1 to 6. A depth scale was used in the tibial trials and molds to determine the adequate thickness of the tibial spacers.

### 2.2. Patients

The study was approved by the local institutional review board, and all study participants provided informed consent. The study population was retrospectively derived from a database and included 278 adult patients with chronic PKI, based on the Musculoskeletal Infection Society criteria [13], who underwent a two-stage revision with PS spacers between January 2015 and March 2022, with a minimum follow-up of 1 year. Those with incomplete radiographic data, PKIs after primary TKA with highly constrained prostheses (e.g., NexGen Legacy Constrained Condylar or Rotating Hinge Knees), PKIs after revision TKA, fungal or tuberculous PKIs, above-knee amputation or death during the spacer period, spacer period of more than 1 year, or permanent spacer retention were excluded from the study. Consequently, there were 203 included patients.

All 203 surgeries were performed by eight fellow-trained arthroplasty surgeons in one center in order to ensure similar approaches to the surgery. The patients were divided into two consecutive cohorts on the basis of the annualized volume of RAs performed by the surgeon, which was obtained by dividing the total number of RA performed by a given surgeon per year by the number of years in which a surgeon performed at least one RA [14]. The estimates were then inspected, and surgeon cutoff points were chosen to divide the patients into two approximately equal volume-based medians. In other words, there were approximately 100 patients in each cohort. Therefore, high volume was defined as ≥14 RAs/year, and low volume was defined as <14 RAs/year [8].

### 2.3. Surgical Technique

All antibiotic-loaded cement spacers were prepared using a 1:5 ratio of antibiotics to bone cement (CMW3; DePuy Synthes, Warsaw, IN, USA). Vancomycin and ceftazidime were routinely used [15]. All spacers were implanted using a routine medial parapatellar approach.

The femoral and tibial trials were sized against the prostheses retrieved after resection, and the corresponding molds were chosen. By applying the femoral trial to the distal femur, the knee joint was positioned at full extension and 90° flexion to determine the adequate thickness of the tibial spacer with a depth scale for gap balance and bony defects. The femoral and tibial spacers were fabricated simultaneously. The femoral spacer was first cemented to the distal femur using an additional package of antibiotic-loaded cement. The tibial spacer was cemented to the proximal tibia after curing the femoral portion. The knee was fully extended with an appropriate alignment to allow the tibial cement to set. Lateral patellar release was performed if necessary. After November 2018, the cam of the femoral spacer was augmented with a K-wire as an endoskeleton to reinforce mechanical strength, according to the study design [8].

### 2.4. Postoperative Protocol

**Figure 1:**
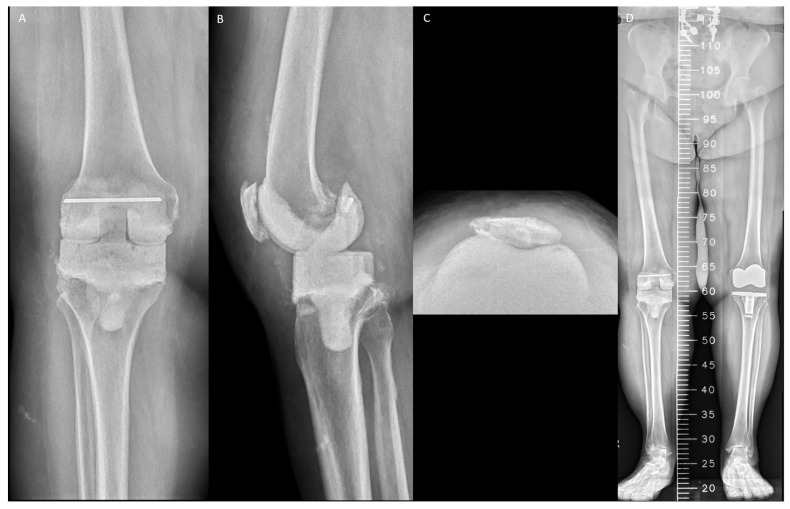
Radiographs of the articulating spacer obtained 1 week after resection arthroplasty: (**A**) standing anteroposterior view; (**B**) standing lateral view; (**C**) merchant view; and (**D**) scanogram of the lower limb.

Radiographic evaluations, including knee weight-bearing anteroposterior, lateral, and Merchant views, and lower-limb scanogram, were conducted 1 week after RA (Figure 1). Radiographs were obtained monthly before reimplantation or when the patient experienced knee pain, swelling, instability, or deformity. Rehabilitation was partial weight-bearing with crutches without a hinged brace. Each patient received at least 4 weeks of organism-specific intravenous antibiotics according to the recommendations of an infection consultant. Oral antibiotics were maintained until C-reactive protein levels and erythrocyte sedimentation rates decreased.

The criteria for reimplantation included negative clinical signs of infection, normalized C-reactive protein and erythrocyte sedimentation rates, and negative arthrocentesis culture after a 2-week antibiotic holiday, which was defined as the period off antibiotics prior to reimplantation [16].

### 2.5. Evaluation

Demographic patient data, including medical comorbidities, microorganisms, spacer information, and spacer period, were extracted from medical charts. The following radiograph findings before reimplantation were recorded as mechanical complications using the INFINITT Picture Archiving and Communications System (INFINITT, Seoul, South Korea): spacer fracture, spacer migration, periprosthetic fracture, joint subluxation/dislocation, or extensor mechanism disruption [10,12,17]. Those with the progression of radiolucent lines around spacers without the above complications were excluded. Mixed complications were counted only as one major complication for each patient (e.g., spacer migration followed by joint dislocation). A musculoskeletal radiologist and two arthroplasty surgeons independently assessed and recorded all radiographic data.

Reoperations for mechanical complications, such as open reduction and partial or total spacer exchange, and extensor mechanism repair data were extracted from operation records; unexpected early reimplantation was also recorded. However, procedures for persistent infections, such as debridement, arthrotomy, and spacer exchange, were not included. Patients with simultaneous complications of mechanical and persistent infections were excluded from the reoperation count.

**Figure 2:**
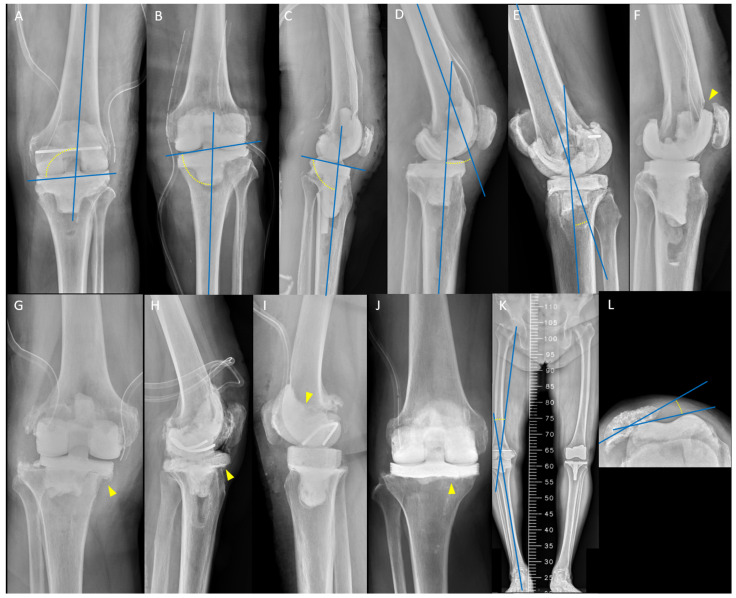
Malalignment and malposition of articulating spacer: (**A**) medial distal femoral angle, 100°; (**B**) medial proximal tibial angle, 83°; (**C**) posterior tibial slope angle, 96°; (**D**) flexion contracture, 26°; (**E**) recurvatum deformity, 11°; (**F**) femoral spacer notching (arrowhead); (**G**) medial overhang of tibial spacer (arrowhead); (**H**) anterior overhang of tibial spacer (arrowhead); (**I**) femoral spacer without cement stem extension (arrowhead); (**J**) tibial spacer without cement stem extension (arrowhead); (**K**) severe varus deformity with hip–knee–ankle angle, 16°; and (**L**) maltracking with lateral patellar tilt, 14°.

Spacer malalignment was defined as medial distal femoral angle <2° or >8° of valgus [18] (Figure 2A), medial proximal tibial angle (PTA) <−3° or >3° deviation from neutral [19] (Figure 2B), posterior tibial slope angle <0° or >7° of flexion [20] (Figure 2C), flexion contracture >15° of flexion [21] (Figure 2D), and recurvatum deformity >5° of extension [22] (Figure 2E); spacer malposition such as anterior femoral notching >3 mm in depth [23] (Figure 2F), and mediolateral or anteroposterior overhang of tibial spacers >3 mm [24] (Figure 2G,H). Cementing technique of spacers with or without cement stem extension was also recorded (Figure 2I,J). All the above data were measured on anteroposterior and lateral short-leg radiographs obtained 1 week after RA [25]. Lower-limb malalignment was defined as a hip–knee–ankle angle <−10° (severe varus) or >10° (severe valgus) on a scanogram [26] (Figure 2K). Patellar maltracking was defined as a lateral patellar tilt >10° on the Merchant view [27] (Figure 2L). Data were measured separately by the same three physicians, and the mean value was calculated. Mixed outliers were also recorded for each patient.

### 2.6. Data Analyses

Data analyses were performed using the Statistical Package for the Social Sciences version 24.0 (IBM Corp., Armonk, NY, USA). Descriptive statistics are presented as means and 95% confidence intervals for continuous variables and as counts and percentages for categorical variables. Differences between two continuous variables were assessed using the Student’s *t*-test, a statistical test used to determine if the means of two groups significantly differ from each other. Differences between two categorical variables were assessed using Fisher’s exact test, a statistical test used to determine if the proportions of categories in two groups significantly differ from each other.

The reliability of mechanical complications was examined using the intraclass correlation coefficient (ICC). ICC is usually found to have a value between 0 and 1. An ICC value below 0.5 is regarded as a sign of poor reliability. On the other hand, an ICC value above 0.9 is regarded as a sign of excellent reliability. Associations between covariates and mechanical complications were determined with univariate logistic regression analysis. Covariates with statistically significant association on univariate analysis were included in a multivariable logistic regression model. Statistical significance was set at *p* < 0.05.

## 3. Results

**Figure 3:**
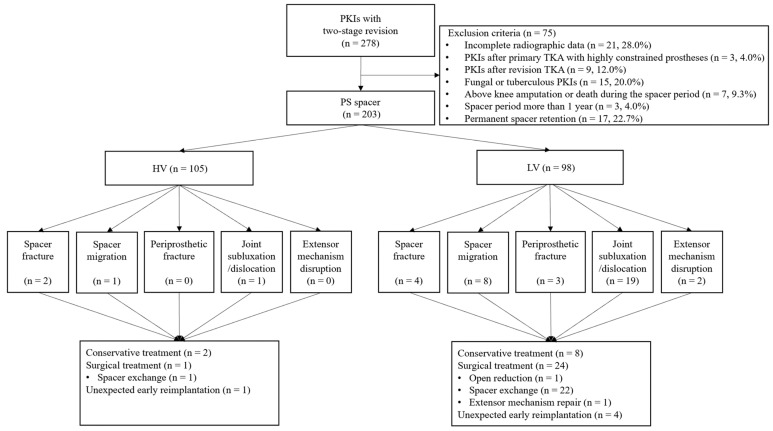
Strengthening the Reporting of Observational Studies in Epidemiology flowchart detailing the design of the study. PKIs, prosthetic knee infections; TKA, total knee arthroplasty; HV, high volume; LV, low volume.

Of the 203 included patients, 105 and 98 were treated by two HV and six LV surgeons, respectively. A single knee was used for each patient. The mean spacer duration was 14.3 weeks. The average follow-up time was 54.1 months (minimal and maximal were 13 and 79 months, respectively). Figure 3 presents the Strengthening the Reporting of Observational Studies in Epidemiology flowchart detailing the study design. There were no significant differences in the demographic data between patients who underwent HV or LV surgery (Table 1).

**Figure 4:**
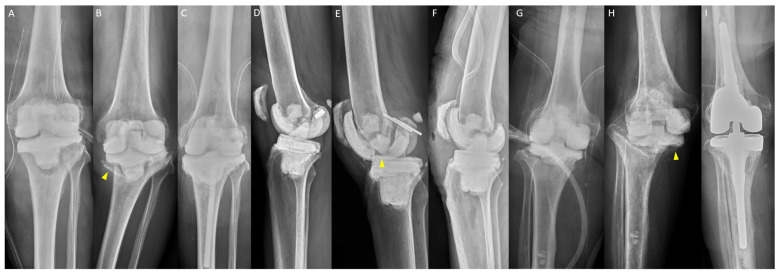
Examples of mechanical complication: (**A**) a 68-year-old man after resection arthroplasty (RA) with varus malalignment of tibial spacer (medial proximal tibial angle, 83°); (**B**) periprosthetic tibial fracture (arrowhead) 3 weeks after spacer insertion; (**C**) reoperation with tibial spacer exchange; (**D**) a 63-year-old man after RA with recurvatum deformity (12°); (**E**) femoral spacer fracture (arrowhead) 4 weeks after spacer insertion; (**F**) reoperation with both spacers exchange; (**G**) a 76-year-old woman after RA with tibial spacer without cement stem extension; (**H**) tibial spacer migration (arrowhead) 6 weeks after spacer insertion; and (**I**) unexpected early reimplantation smoothly.

There were 40 (19.7%) mechanical complications in 203 spacers. The ICC value for mechanical complications was 0.974 (range, 0.918 to 0.990; *p* < 0.001). The outcomes of spacer placement performed by HV or LV surgeons are presented in Table 2. The overall mechanical complication rate was lower in HV surgeons (3.8%) than in LV surgeons (36.7%) (*p* < 0.001), especially for joint subluxation or dislocation. The overall reoperation rate was lower in HV surgeons (0.9%) than in LV surgeons (24.5%) (*p* < 0.001), especially in spacer exchanges (Figure 4A–F). Among the cases that had 36 mechanical complications in LV surgeons, 66.7% (24/36) underwent reoperation, and 91.7% (22/24) of the reoperation procedures were spacer exchanges. There were 1 (0.9%) and 4 (4.1%) knees that had unexpected early reimplantation that were performed smoothly after mechanical complications by HV and LV surgeons, respectively (Figure 4G–I). Table 3 presents the details of these mechanical complications, in which all mechanisms were atraumatic and 92.5% had early failure (≤6 weeks) after spacer insertion. All knees requiring reoperation were treated with hinge braces and were free of instability before reimplantation.

Moreover, LV spacers were found to be associated with an increased requirement for highly constrained prostheses, such as rotating hinge knees (LV vs. HV, 17.3% vs. 0.9%, *p* = 0.008), and these knees had mechanical complications during the spacer period. Additionally, 47.2% (17/36) of patients required hinge knees after mechanical spacer failure. Table 4 presents the univariate risk factors for mechanical complications. The multivariate logistic regression analysis identified PTA < 87°, recurvatum deformity, and tibial spacer use without a cement stem extension as independent risk factors for mechanical failure (Table 5).

## 4. Discussion

This study compared the mechanical complications in RA with articulating spacers according to surgeon volume and analyzed the risk factors. Our results showed that HV surgeons have a lower risk of overall mechanical complications and reoperations. The mechanical complications increased the level of constraint in the final revision of knee arthroplasty. The identified risk factors for mechanical failure were varus malalignment of the tibial spacer, recurvatum deformity, and tibial spacer without cement stem extension.

Tan et al. and George et al. reported that the rates of spacer mechanical complications were approximately 17 to 18% [28,29], and our rate, 19.7%, was consistent with these data. Our mechanical complications were all atraumatic, with the majority (92.5%) of spacers failing at <6 weeks, which might be related to the unstable biomechanical environment in PKI after RA [12], in addition to the weaker strength of the cement due to high-dose antibiotics [8], and the spacers were fixed in the bone ends without tight interdigitation for ease of removal with minimal bone loss [17]. Lin et al. found that a low surgical volume was a risk factor for spacer fractures with dislocation (odds ratio, 8.13) [8]. Moreover, better infection control and reimplantation rates have been reported in patients treated at specialized PKI centers [11]. Superior results have been found for the treatment at subspecialty centers of bone and joint infections [30]. Although our institution performed protocol-driven RAs with articulating spacers, the clinical results were different after analysis by surgical volume; HV surgeons had only 3.8% mechanical complications compared with 36.7% in LV surgeons. This discrepancy might be related to the learning curve or other associated risk factors, as LV surgeons only performed 4.23 RAs/year (range, 1.9 to 8.6 RAs/year), compared with HV surgeons, who treated multiple referred PKIs per month and performed many RAs annually.

Lau et al. found that 34.9% (22/63) of coronal subluxations of the articulating spacers required highly constrained knee systems during reimplantation [7]. Lin et al. reported that 70% (14/20) of dislocated spacers needed rotating hinge knees during reimplantation and proposed the concept “added insult to injury” when knee dislocations occur after RA with articulating spacers, suggesting that surgeons must strive to prevent joint dislocation during the spacer period [12]. Lin et al. also mentioned that to prevent post-cam failure of the PS spacer, an endoskeleton-reinforced femoral cam or additional hinged brace protection should be considered [8]. The finding that hinge knees were required in 47.2% of patients after mechanical spacer failure in our LV group is substantial, as this illustrates that the mechanical complications increased the level of constraint in final revision knee arthroplasty [12]. The above findings indicate that soft tissue compromise after an unstable joint event might be a predictor for highly constrained revision prostheses, which should be prepared and made available at the time of reimplantation. In two-stage revisions, Struelens et al. reported that only 43% of 154 articulating spacers were considered to be optimally sized and positioned, and mechanical complications were as high as 57% [6]. Lin et al. proposed that malalignment and malrotation of femoral and tibial spacers may lead to spacer overstress [8]. Our PTA <87° tibial spacers had a 5.3 times higher risk of mechanical complications. Medial tibial plateau bony loss or tibial bowing may lead to the varus placement of the tibial spacer [31]. Gililland et al. reported a useful technique to restore a stable bony tibial platform perpendicular to the mechanical axis by using a standard extramedullary tibial cutting jig and drop rod to avoid malalignment of the tibial spacer [32]. From our HV surgeon’s experience, cement augmentation for bony defects is a promising technique if there is huge bone loss after the cut or if the joint line is too low.

Recurvatum deformity is the rarest form of instability after TKA and is difficult to correct [33]. Many studies have reported the use of rotating hinge knees as extension stops to treat post-TKA recurvatum [34]. The etiologies of pre-TKA and range of motion pre-RA in our cohort were not fully documented because of the multiple surgeons involved in the current study. However, multiple surgeries, including RA, might result in weak quadriceps and compromise of the extensor mechanism locking during the stance phase of gait, which adds to the risk of recurvatum, as hyperextension helps stabilize the knee [35]. A huge bony defect, insufficient collateral ligaments, and compromised posterior capsule during infection and after RA might result in joint laxity, even with a spacer [8,12,17]. Moreover, the post-cam mechanism of the PS spacer cannot resist the force during knee hyperextension; thus, mechanical complications occur. Although surgeons have used a depth scale within tibial trials and molds to ensure accurate thickness of the tibial spacer to fulfill the gap balance, there is still a learning curve during spacer fabrication and implantation [8]. Based on our HV surgeon’s experience, careful examination of the ligament status during RA and placement of the knee with a spacer in slight flexion will avoid recurvatum complications.

In revision TKA, Oh et al. reported that metaphyseal fixation is important for ensuring the stability of the component [36]; Lee et al. recommended the use of an extension stem with a tibial canal filling ratio > 0.85 to minimize tibial component loosening [37]. Jung et al. reported that the spiked bottom of the tibial spacer provides superior stability and lowers spacer translation and tilting than a flat bottom [38]. Tsai et al. used the bottom of a tibial spacer with a cement stem extension to fill the metaphyseal defect and stably implant a spacer to minimize spacer loosening and migration [17]. Gililland et al. suggested a stemmed spacer with a cement dowel for increased antibiotic elution and added stable fixation [32]. We found that a femoral spacer without a cement stem extension was not associated with mechanical failure, suggesting a difference in bony end morphology and kinematics between the distal femur and the proximal tibia [8]. Our tibial spacers without cement stem extensions had a 7.25 times higher risk of mechanical complications, indicating that the stem plays an important role in additional stability. We strongly suggest using cement stem extensions of the tibial spacer to avoid spacer dislodgement.

This study has some limitations. This was a retrospective cohort study involving only one center. A higher-powered, prospective, controlled study with multiple centers is needed to confirm the effect of surgical volume. The physical activity levels of patients were not measured, and detailed chart records of every non-traumatic event were lacking, but there was no clear association between mechanical failure and more active patients [39]. The lack of computer tomography prevented spacer malrotation measurements, which may have caused loosening and further failure [40]. We did not evaluate the effect of spacers malfabricated by LV surgeons, which might increase the risk of mechanical complications [8].

Nevertheless, this was the first study to directly compare surgical volume in RA and radiographic measurements of articulating spacers with a large sample size and sufficient power to detect effects. Our results broadly indicate better outcomes for HV surgeons than for LV surgeons. This finding provides a further research platform for establishing a new subspecialty for prosthetic joint infection with experienced HV surgeons to improve the outcomes of two-stage revision.

## 5. Conclusions

This was the first retrospective study to compare the effect of surgeon volume on mechanical complications after RA with an articulating spacer in a two-stage revision of PKI. Based on the findings, we made the following three conclusions: (1) HV surgeons had a lower rate of mechanical complications and reoperations than LV surgeons; (2) mechanical complications increased the level of constraint in the final revision of knee arthroplasty; and (3) all surgeons should avoid tibial spacer varus malalignment and recurvatum deformity and always use a cement stem extension with a tibial spacer.

## Figures and Tables

**Table 1:** Demographic data of patients performed on by HV or LV surgeons.

Variables	Surgeon Volume		*p*-Value
	HV (n = 105)	LV (n = 98)	
Age (years)	67.4 (59–83)	66.1 (58–82)	0.713
Sex (female)	59 (56.2)	60 (61.2)	0.554
BMI (kg/m^2^)	27.3 (21.0–34.6)	28.6 (21.7–33.2)	0.676
Right laterality	54 (51.4)	46 (46.9)	0.262
Constraint of the original prosthesis			
Cruciate-retaining	37 (35.2)	35 (35.7)	0.906
Posterior-stabilized	68 (64.8)	63 (64.3)	0.894
Smoking status			
Never-smokers	49 (46.7)	42 (42.9)	0.648
Current/ex-smokers	56 (53.3)	56 (57.1)	0.517
Insurance status			
Insured	104 (99.0)	98 (100.0)	0.930
Uninsured	1 (1.0)	0 (0.0)	0.964
Socioeconomic status			
Low	42 (40.0)	39 (39.8)	0.911
Middle	38 (36.2)	37 (37.8)	0.890
High	25 (23.8)	22 (22.4)	0.835
Charlson Comorbidity Index			
0	52 (49.5)	47 (48.0)	0.876
1–2	38 (36.2)	36 (36.7)	0.937
3+	15 (14.3)	15 (15.3)	0.882
McPherson host grade			
Uncompromised	51 (48.6)	45 (45.9)	0.704
Compromised	30 (28.5)	29 (29.6)	0.816
Substantially compromised	24 (22.9)	24 (24.5)	0.773
Microorganisms			
*Staphylococcus* species	35 (33.3)	33 (33.7)	0.938
*Streptococcus* species	20 (19.0)	18 (18.4)	0.863
Gram-positive cocci	15 (14.3)	14 (14.3)	0.999
Gram-negative species	16 (15.2)	17 (17.3)	0.803
Polymicrobial	8 (7.6)	8 (8.2)	0.830
Culture negative	11 (10.5)	8 (8.2)	0.709
Spacer information			
Femoral spacer size (No.)	3.9 (2–5)	3.8 (2–5)	0.774
Femoral spacer with endoskeleton	52 (49.5)	47 (48.0)	0.808
Tibial spacer size (No.)	4.0 (2–5)	3.9 (2–5)	0.822
Tibial spacer thickness (mm)	13.2 (10–19)	13.7 (10–21)	0.762
Spacer period (weeks)	14.6 (11–24)	14.4 (10–23)	0.825
Follow-up period (months)	54.7 (13–79)	53.6 (13–77)	0.692

Values are presented as means [95% confidence intervals] or n (%). HV, high volume; LV, low volume; BMI, body mass index; No., number.

**Table 2:** Outcomes of PKIs performed by HV and LV surgeons.

Parameters	Surgeon Volume		*p*-Value
	HV (n = 105)	LV (n = 98)	
Overall mechanical complication	4 (3.8)	36 (36.7)	**<0.001**
Spacer fracture	2 (1.9)	4 (4.1)	0.284
Spacer migration	1 (0.9)	8 (8.2)	0.051
Periprosthetic fracture	0 (0.0)	3 (3.1)	0.417
Joint subluxation/dislocation	1 (0.9)	19 (19.4)	**0.006**
Extensor mechanism disruption	0 (0.0)	2 (2.0)	0.596
Overall reoperation	1 (0.9)	24 (24.5)	**<0.001**
Open reduction	0 (0.0)	1 (1.0)	0.891
Spacer exchange	1 (0.9)	22 (22.4)	**0.003**
Extensor mechanism repair	0 (0.0)	1 (1.0)	0.891
Unexpected early reimplantation	1 (0.9)	4 (4.1)	0.110
Constraint of reimplantation prosthesis			
Legacy constrained condylar knee	104 (99.1)	81 (82.7)	**0.007**
Rotating hinge knee	1 (0.9)	17 (17.3)	**0.008**

Values are presented as n (%). *p*-values in bold are statistically significant. PKI, prosthetic knee infection; HV, high volume; LV, low volume.

**Table 3:** Details of patients with mechanical complications by HV and LV surgeons.

Patient No.	Age (Years)	Sex	Radiographic Outlier	Mechanical Complication	Timing after Spacer (Weeks)	Intervention
HV surgeons						
1	69	F	PTA < 87°; HKA < −10°	TS migration	3	Hinge knee brace
2	64	M	TS overhang; DFA > 98°	TS fracture	4	Hinge knee brace
3	76	M	PTA > 90°; FS notching	TS fracture	5	Both spacers exchange
4	77	F	Recurvatum deformity	Joint dislocation	6	Unexpected early reimplantation
LV surgeons						
1	73	M	PTA < 87°; DFA < 92°	TS fracture	2	Hinge knee brace
2	66	M	PTA < 87°; DFA > 98°	Joint subluxation	5	Hinge knee brace
3	82	M	PTA < 87°; FS notching	Joint subluxation	4	Hinge knee brace
4	63	F	PTA < 87°; patellar maltracking	Joint dislocation	4	Open reduction
5	66	F	PTA < 87°; HKA < −10°	Joint subluxation	3	TS exchange
6	86	F	PTA < 87°; flexion contracture	Joint dislocation	6	TS exchange
7	77	F	PTA < 87°	Joint dislocation	4	TS exchange
8	71	M	PTA < 87°; FS without cement stem extension	Joint dislocation	5	TS exchange
9	68	M	PTA < 87°	Periprosthetic tibial fracture	3	TS exchange
10	73	M	PTA < 87°; flexion contracture	Periprosthetic tibial fracture	4	Both spacers exchange
11	72	F	PTA < 87°; HKA < −10°	Both spacers fracture	6	Unexpected early reimplantation
12	68	F	Recurvatum deformity; DFA > 98°	Joint subluxation	5	Hinge knee brace
13	58	M	Recurvatum deformity	Joint subluxation	7	Hinge knee brace
14	72	M	Recurvatum deformity; TS without cement stem extension	Joint dislocation	4	Both spacers exchange
15	80	F	Recurvatum deformity; TS overhang	Joint dislocation	6	Both spacers exchange
16	66	F	Recurvatum deformity; HKA angle < −10°	Joint dislocation	7	FS exchange
17	60	M	Recurvatum deformity; DFA < 92°	Joint dislocation	4	FS exchange
18	80	F	Recurvatum deformity; patellar maltracking	Joint dislocation	8	FS exchange
19	67	F	Recurvatum deformity; FS notching	Joint dislocation	4	FS exchange
20	63	M	Recurvatum deformity	FS fracture	4	Both spacers exchange
21	60	M	Recurvatum deformity; FS without cement stem extension	Periprosthetic tibial fracture	6	Unexpected early reimplantation
22	61	M	TS without cement stem extension	Joint subluxation	4	Hinge knee brace
23	73	F	TS without cement stem extension; DFA > 98°	Joint subluxation	5	Hinge knee brace
24	61	M	TS without cement stem extension	Joint dislocation	3	Both spacers exchange
25	72	M	TS without cement stem extension; DFA < 92°	Joint dislocation	6	Both spacers exchange
26	75	F	TS without cement stem extension; patellar maltracking	TS migration	3	TS exchange
27	68	F	TS without cement stem extension	TS migration	4	TS exchange
28	69	M	TS without cement stem extension; PSA > 90°	TS migration	4	Both spacers exchange
29	73	F	TS without cement stem extension; TS overhang	TS migration	3	TS exchange
30	60	F	TS without cement stem extension; HKA angle < −10°	TS migration	5	TS exchange
31	66	M	TS without cement stem extension; PTA > 93°	TS migration	3	TS exchange
32	76	F	TS without cement stem extension	TS migration	6	Unexpected early reimplantation
33	77	F	TS overhang; DFA > 98°	Extensor mechanism disruption	3	TS exchange
34	67	M	Patellar maltracking; DFA < 92°	Extensor mechanism disruption	4	Lateral release
35	64	F	HKA < −10°; DFA < 92°	TS migration	6	Unexpected early reimplantation
36	72	F	HKA < −10°; flexion contracture	Both spacers fracture	3	Hinge knee brace

HV, high volume; LV, low volume; No., number; PTA, medial proximal tibial angle; HKA, hip–knee–ankle; TS, tibial spacer; PSA, posterior tibial slope angle; FS, femoral spacer; DFA, medial distal femoral angle.

**Table 4:** Univariate analysis for risk factors associated with mechanical complications.

Variables	No Mechanical Complications (n = 163)	Mechanical Complications (n = 40)	Odds Ratio (95% CI)	*p*-Value
Age ≥ 65 years	128 (78.5)	30 (75.0)	0.91 (0.19–2.43)	0.638
Sex (female)	98 (60.1)	21 (52.5)	0.87 (0.12–1.92)	0.507
BMI ≥ 25.0 kg/m^2^	96 (58.9)	28 (70.0)	4.43 (1.84–23.5)	**0.038**
Right laterality	77 (47.2)	23 (57.5)	1.99 (0.78–5.37)	0.059
Constraint of the original prosthesis				
Cruciate-retaining	57 (35.0)	15 (37.5)	1.02 (0.30–3.84)	0.710
Posterior-stabilized	106 (65.0)	25 (62.5)	0.90 (0.44–2.89)	0.569
Smoking status				
Never-smokers	73 (44.8)	18 (45.0)	1.00 (0.18–1.76)	0.996
Current/ex-smokers	90 (52.4)	22 (55.0)	1.02 (0.11–3.03)	0.834
Insurance status				
Insured	162 (99.4)	40 (100.0)	1.01 (0.33–1.68)	0.914
Uninsured	1 (0.6)	0 (0.0)	0.99 (0.22–1.37)	0.939
Socioeconomic status				
Low	64 (39.3)	17 (42.5)	1.09 (0.40–4.13)	0.456
Middle	61 (37.4)	14 (35.0)	0.93 (0.13–2.10)	0.741
High, n (%)	38 (23.3)	9 (22.5)	0.98 (0.38–2.31)	0.853
Charlson Comorbidity Index				
0	81 (49.7)	18 (45.0)	0.91 (0.51–2.27)	0.727
1–2	59 (36.2)	15 (37.5)	1.02 (0.52–2.63)	0.734
3+	23 (14.1)	7 (17.5)	1.03 (0.49–3.11)	0.515
McPherson host grade				
Uncompromised	79 (48.5)	17 (42.5)	0.89 (0.28–2.16)	0.760
Compromised	47 (28.8)	12 (30.0)	1.09 (0.35–4.61)	0.672
Substantially compromised	37 (22.7)	11 (27.5)	1.11 (0.59–5.48)	0.518
Microorganisms				
*Staphylococcus* species	54 (33.1)	14 (35.0)	1.07 (0.39–3.44)	0.630
*Streptococcus* species	31 (19.0)	7 (17.5)	0.98 (0.20–2.11)	0.832
Gram-positive cocci	24 (14.7)	5 (12.5)	0.97 (0.21–2.09)	0.814
Gram-negative species	27 (16.6)	6 (15.0)	0.98 (0.23–2.07)	0.827
Polymicrobial	13 (7.9)	3 (7.5)	1.00 (0.12–1.97)	0.964
Culture negative	14 (8.6)	5 (12.5)	1.12 (0.13–6.40)	0.633
Spacer information				
Femoral spacer size ≤ 2	76 (46.6)	23 (57.5)	3.97 (1.19–12.3)	**0.042**
Femoral spacer with endoskeleton	81 (49.7)	18 (45.0)	0.91 (0.48–2.33)	0.718
Tibial spacer size ≤ 2	73 (44.8)	21 (52.5)	3.19 (1.28–10.6)	0.051
Tibial spacer thickness ≥ 13 mm	79 (48.5)	22 (55.0)	3.04 (1.21–10.3)	0.053
Spacer malalignment and malposition				
DFA > 98°	18 (11.0)	5 (12.5)	1.02 (0.38–3.20)	0.863
DFA < 92°	11 (6.7)	5 (12.5)	3.03 (1.19–9.73)	0.056
PTA > 93°	8 (4.9)	1 (2.5)	0.90 (032–2.00)	0.778
PTA < 87°	15 (9.2)	12 (30.0)	6.76 (2.88–43.3)	**0.005**
PSA > 90°	7 (4.3)	2 (5.0)	1.01 (0.76–1.86)	0.901
PSA < 83°	2 (1.2)	0 (0.0)	0.98 (0.63–1.69)	0.806
Flexion contracture	15 (9.2)	3 (7.5)	0.97 (0.23–1.98)	0.716
Recurvatum deformity	9 (5.5)	11 (27.5)	7.31 (2.04–50.7)	**0.003**
FS notching	17 (10.4)	3 (7.5)	0.95 (0.51–1.87)	0.706
TS overhang	26 (15.9)	5 (12.5)	0.91 (0.28–1.83)	0.739
FS without cement stem extension	18 (11.0)	2 (5.0)	0.72 (0.13–1.76)	0.132
TS without cement stem extension	7 (4.3)	12 (30.0)	8.94 (2.76–62.6)	**<0.001**
HKA angle > 10°	2 (1.2)	0 (0.0)	0.98 (0.20–2.02)	0.870
HKA angle < −10°	10 (6.1)	7 (17.5)	4.11 (2.24–22.1)	**0.041**
Patellar maltracking	23 (14.1)	4 (10.0)	0.94 (1.11–2.04)	0.703
Spacer period (weeks)	14.1 (12–24)	14.9 (11–22)	1.11 (0.48–3.78)	0.529
Follow-up period (months)	54.2 (13–78)	53.9 (14–75)	0.97 (0.46–2.32)	0.631

Values are presented as n (%) and means [95% confidence intervals]. *p*-values in bold are statistically significant. BMI, body mass index; DFA, medial distal femoral angle; PTA, medial proximal tibial angle; PSA, posterior tibial slope angle; FS, femoral spacer; TS, tibial spacer; HKA, hip–knee–ankle; CI, confidence interval.

**Table 5:** Multivariate analysis for risk factors associated with mechanical complications.

Variables	Adjusted Odds Ratio (95% CI)	*p*-Value
BMI ≥ 25.0 kg/m^2^	3.01 (1.32–12.4)	0.054
Femoral spacer size ≤ 2	2.63 (1.04–9.18)	0.127
Medial PTA < 87°	5.30 (2.11–37.6)	**0.009**
Recurvatum deformity	6.43 (1.93–41.4)	**0.007**
TS without cement stem extension	7.25 (2.24–53.5)	**0.002**
HKA angle < −10°	2.99 (1.62–11.4)	0.075

*p*-values in bold are statistically significant. CI, confidence interval; BMI, body mass index; PTA, proximal tibial angle; TS, tibial spacer; HKA, hip–knee–ankle.

## Data Availability

The authors declare that all data supporting the findings of this study are available within the article.

## References

[1] DeBoer D.K. (2020). Comparison of Traditional Molded, First-Generation Premolded, and Second-Generation Premolded Antibiotic-Loaded Polymethylmethacrylate Articulating Spacers for Treatment of Chronic Prosthetic Joint Infection of the Knee. J. Arthroplast..

[2] Magruder M.L., Yao V.J.H., Rodriguez A.N., Ng M.K., Piuzzi N.S., Mont M.A. (2024). History of Diabetic Foot Ulcer is Associated With Increased Risk of Prosthetic Joint Infection and Sepsis After Total Joint Arthroplasty. J. Arthroplast..

[3] Piple A.S., Wang J.C., Kebaish K.J., Mills E.S., Oakes D.A., Lieberman J.R., Christ A.B., Heckmann N.D. (2023). Does Prednisone Dose Affect Rates of Periprosthetic Joint Infection Following Primary Total Hip and Total Knee Arthroplasty?. J. Arthroplast..

[4] Florance J., Chang J., Kelly P.J., Smith D., Bolognesi M., Seyler T., Ryan S.P. (2024). Inferior Outcomes for Patients Transferred Between Surgical Stages for Knee Periprosthetic Joint Infection. J. Arthroplast..

[5] Nahhas C.R., Chalmers P.N., Parvizi J., Sporer S.M., Berend K.R., Moric M., Chen A.F., Austin M.S., Deirmengian G.K., Morris M.J. (2020). A Randomized Trial of Static and Articulating Spacers for the Treatment of Infection Following Total Knee Arthroplasty. J. Bone Jt. Surg. Am..

[6] Struelens B., Claes S., Bellemans J. (2013). Spacer-related problems in two-stage revision knee arthroplasty. Acta Orthop. Belg..

[7] Lau A.C., Howard J.L., Macdonald S.J., Teeter M.G., Lanting B.A. (2016). The Effect of Subluxation of Articulating Antibiotic Spacers on Bone Defects and Degree of Constraint in Revision Knee Arthroplasty. J. Arthroplast..

[8] Lin T.L., Tsai C.H., Fong Y.C., Shie M.Y., Chen H.Y., Chen Y.W. (2022). Posterior-Stabilized Antibiotic Cement Articulating Spacer with Endoskeleton-Reinforced Cam Reduces Rate of Post-Cam Mechanical Complications in Prosthetic Knee Infection: A Preliminary Study. J. Arthroplast..

[9] Wilson S., Marx R.G., Pan T.J., Lyman S. (2016). Meaningful Thresholds for the Volume-Outcome Relationship in Total Knee Arthroplasty. J. Bone Jt. Surg. Am..

[10] Roof M.A., Sharan M., Merkow D., Feng J.E., Long W.J., Schwarzkopf R.S. (2021). High-volume revision surgeons have better outcomes following revision total knee arthroplasty. Bone Jt. J..

[11] Fehring T.K., Otero J.E., Curtin B.M., Fehring K.A., Metcalf R., Rowe T.M., Springer B.D. (2023). Does Treatment at a Specialized Prosthetic Joint Infection Center Improve the Rate of Reimplantation?. J. Arthroplast..

[12] Lin T.L., Tsai C.H., Fong Y.C., Shie M.Y., Chen H.Y., Chen Y.W. (2021). Cruciate-Retaining vs Posterior-Stabilized Antibiotic Cement Articulating Spacers for Two-Stage Revision of Prosthetic Knee Infection: A Retrospective Cohort Study. J. Arthroplast..

[13] Parvizi J., Gehrke T. (2014). Definition of periprosthetic joint infection. J. Arthroplast..

[14] Wright J.D., Hershman D.L., Burke W.M., Lu Y.-S., Neugut A.I., Lewin S.N., Herzog T.J. (2012). Influence of surgical volume on outcome for laparoscopic hysterectomy for endometrial cancer. Ann. Surg. Oncol..

[15] Hsu Y.H., Hu C.C., Hsieh P.H., Shih H.N., Ueng S.W., Chang Y. (2017). Vancomycin and Ceftazidime in Bone Cement as a Potentially Effective Treatment for Knee Periprosthetic Joint Infection. J. Bone Jt. Surg. Am..

[16] Diaz-Ledezma C., Higuera C.A., Parvizi J. (2013). Success after treatment of periprosthetic joint infection: A Delphi-based international multidisciplinary consensus. Clin. Orthop. Relat. Res..

[17] Tsai C.-H., Hsu H.-C., Chen H.-Y., Fong Y.-C., Ho M.-W., Chou C.-H., Chen Y.-W., Shie M.-Y., Lin T.-L. (2019). A preliminary study of the novel antibiotic-loaded cement computer-aided design-articulating spacer for the treatment of periprosthetic knee infection. J. Orthop. Surg. Res..

[18] Ritter M.A., Davis K.E., Meding J.B., Pierson J.L., Berend M.E., Malinzak R.A. (2011). The effect of alignment and BMI on failure of total knee replacement. J. Bone Jt. Surg. Am..

[19] Abane L., Anract P., Boisgard S., Descamps S., Courpied J.P., Hamadouche M. (2015). A comparison of patient-specific and conventional instrumentation for total knee arthroplasty: A multicentre randomised controlled trial. Bone Jt. J..

[20] Kim Y.H., Park J.W., Kim J.S., Park S.D. (2014). The relationship between the survival of total knee arthroplasty and postoperative coronal, sagittal and rotational alignment of knee prosthesis. Int. Orthop..

[21] Song S.J., Lee H.W., Park C.H. (2023). Predictors of Recurrent Flexion Contracture after Total Knee Arthroplasty in Osteoarthritic Knees with Greater Than 15° Flexion Contracture. Clin. Orthop. Surg..

[22] Shagawa M., Maruyama S., Sekine C., Yokota H., Hirabayashi R., Hirata A., Yokoyama M., Edama M. (2021). Comparison of anterior knee laxity, stiffness, genu recurvatum, and general joint laxity in the late follicular phase and the ovulatory phase of the menstrual cycle. BMC Musculoskelet. Disord..

[23] Puranik H.G., Mukartihal R., Patil S.S., Dhanasekaran S.R., Menon V.K. (2019). Does Femoral Notching During Total Knee Arthroplasty Influence Periprosthetic Fracture. A Prospective Study. J. Arthroplast..

[24] Nielsen C.S., Nebergall A., Huddleston J., Kallemose T., Malchau H., Troelsen A. (2018). Medial Overhang of the Tibial Component Is Associated With Higher Risk of Inferior Knee Injury and Osteoarthritis Outcome Score Pain After Knee Replacement. J. Arthroplast..

[25] Tammachote N., Kriengburapha N., Chaiwuttisak A., Kanitnate S., Boontanapibul K. (2018). Is Regular Knee Radiograph Reliable Enough to Assess the Knee Prosthesis Position?. J. Arthroplast..

[26] Thienpont E., Parvizi J. (2016). A New Classification for the Varus Knee. J. Arthroplast..

[27] Kleimeyer J.P., McQuillan T.J., Arsoy D., Aggarwal V.K., Amanatullah D.F. (2021). Agreement and Reliability of Lateral Patellar Tilt and Displacement following Total Knee Arthroplasty with Patellar Resurfacing. J. Knee Surg..

[28] George J., Miller E.M., Curtis G.L., Klika A.K., Barsoum W.K., Mont M.A., Higuera C.A. (2018). Success of Two-Stage Reimplantation in Patients Requiring an Interim Spacer Exchange. J. Arthroplast..

[29] Tan T.L., Goswami K., Kheir M.M., Xu C., Wang Q., Parvizi J. (2019). Surgical Treatment of Chronic Periprosthetic Joint Infection: Fate of Spacer Exchanges. J. Arthroplast..

[30] Ferry T., Seng P., Mainard D., Jenny J.-Y., Laurent F., Senneville E., Grare M., Jolivet-Gougeon A., Bernard L., Marmor S. (2019). The CRIOAc healthcare network in France: A nationwide Health Ministry program to improve the management of bone and joint infection. Orthop. Traumatol. Surg. Res..

[31] Palanisami D., Jagdishbhai C.P., Manohar M., Ramesh P., Natesan R., Shanmuganathan R. (2019). Improving the accuracy of tibial component placement during total knee replacement in varus knees with tibial bowing: A prospective randomised controlled study. Knee.

[32] Gililland J.M., Carlson V.R., Fehring K., Springer B.D., Griffin W.L., Anderson L.A. (2020). Balanced, Stemmed, and Augmented Articulating Total Knee Spacer Technique. Arthroplast. Today.

[33] Mesnard G., Batailler C., Fary C., Schmidt A., Servien E., Lustig S. (2021). Posterior-Stabilized TKA in Patients with Severe Genu Recurvatum Achieves Good Clinical and Radiological Results at 5-year Minimum Follow-Up: A Case-Controlled Study. J. Arthroplast..

[34] Al-Jabri T., Brivio A., Maffulli N., Barrett D. (2021). Management of instability after primary total knee arthroplasty: An evidence-based review. J. Orthop. Surg. Res..

[35] Cottino U., Sculco P.K., Sierra R.J., Abdel M.P. (2016). Instability After Total Knee Arthroplasty. Orthop. Clin. N. Am..

[36] Oh J.H., Scuderi G.R. (2021). Zonal Fixation in Revision TKA: The Key Is Metaphyseal Fixation. J. Knee Surg..

[37] Lee S.H., Shih H.N., Chang C.H., Lu T.W., Chang Y.H., Lin Y.C. (2020). Influence of extension stem length and diameter on clinical and radiographic outcomes of revision total knee arthroplasty. BMC Musculoskelet. Disord..

[38] Jung K.H., Lee C.C., Kim T.H., Han J.W., Park K.B. (2022). Does spiked tibial cement spacer reduce spacer-related problems in two-stage revision total knee arthroplasty for infection?. Int. Orthop..

[39] Sylvia L.G., Bernstein E.E., Hubbard J.L., Keating L., Anderson E.J. (2014). Practical guide to measuring physical activity. J. Acad. Nutr. Diet..

[40] Yoshino K., Hagiwara S., Nakamura J., Tsukeoka T., Tsuneizumi Y., Ohtori S. (2019). Intra- and interobserver reliability and agreement in three-dimensional computed tomography measurements of component positions after total knee arthroplasty. Knee.

